# Carboxyhemoglobin as biomarker of late-onset sepsis in preterm infants

**DOI:** 10.1007/s00431-023-05120-y

**Published:** 2023-07-27

**Authors:** Carlo Dani, Giulia Remaschi, Nicolò Monti, Simone Pratesi

**Affiliations:** 1 Division of Neonatology, Careggi University Hospital of Florence, Largo Brambilla, 3 - 50141 Florence, Italy; 2 Department of Neurosciences, Psychology, Drug Research and Child Health, University of Florence, Florence, Italy

**Keywords:** Carboxyhemoglobin, Sepsis, Preterm infant

## Abstract

Carboxyhemoglobin (COHb) is considered a biomarker of oxidative stress and previous studies reported an increase in COHb levels in preterm infants who develop late-onset sepsis (LOS). Our aim was to assess the correlation between COHb levels and the risk for LOS development. We retrospectively studied 100 preterm infants, 50 in the LOS and 50 in the no LOS group. COHb levels were measured on the day of diagnosis of the first episode of LOS, 3, 2, and 1 days before and 1 and 4 days after the onset of LOS. Logistic regression analysis showed that a higher level of COHb 2 days before the diagnosis of LOS increases the risk for LOS development (OR 12.150, 95% Cl 1.311–12.605; *P* = 0.028). A COHb level of 1.55% measured 2 days before the diagnosis of LOS is the best predictive threshold for LOS with a sensitivity of 70% and a specificity of 70%.

*    Conclusion*: Increased levels of COHb may predict the diagnosis of LOS in very preterm infants with a good accuracy. If further studies confirm our findings, this easy-to-measure biomarker could provide neonatologists with another tool for monitoring and early diagnosis of sepsis in high-risk patients.

**Table:** 

**What is Known:** *• Carboxyhemoglobin (COHb) is a biomarker of oxidative stress.* *• Previous studies reported an increase in COHb levels in preterm infants who develop late-onset sepsis (LOS).*	
**What is New:** *• COHb levels increased two days before the diagnosis of LOS and this increase was associated with the risk for developing LOS.* *• ROC curve analysis for COHb measured two days before the diagnosis of LOS showed that 1.55% is the best predictive threshold for LOS with a sensitivity of 70% and a specificity of 70%.*

**What is Known:**

*• Carboxyhemoglobin (COHb) is a biomarker of oxidative stress.*

*• Previous studies reported an increase in COHb levels in preterm infants who develop late-onset sepsis (LOS).*

**What is New:**

*• COHb levels increased two days before the diagnosis of LOS and this increase was associated with the risk for developing LOS.*

*• ROC curve analysis for COHb measured two days before the diagnosis of LOS showed that 1.55% is the best predictive threshold for LOS with a sensitivity of 70% and a specificity of 70%.*

## Introduction

Carbon monoxide (CO) is naturally and endogenously produced through many enzymatic and non-enzymatic pathways. Approximately 85% of CO is produced by heme oxygenase (HO), which catabolizes heme to CO, iron, and biliverdin [1]. CO binds competitively to hemoglobin, in preference to oxygen, to form carboxyhemoglobin (COHb).

It has been reported that CO can contribute to the regulation of systemic blood pressure and cardiac function in both physiologic and pathologic conditions, inducing vasodilatation through cGMP pathway. Moreover, CO in non-toxic concentration has also been reported to have a physiological and cytoprotective effect in response to cellular stress in inflammatory and immune disorders [2].

On the other hand, HO increases in response to oxidative stress and inflammation, and high levels of COHb have been detected in oxidative stress related condition, such as sepsis and shock in both adult and pediatric populations. Therefore, it is considered a biomarker of oxidative stress [3–5]. Consistently, some studies have evaluated the possible correlation between blood COHb levels and the risk for developing some severe prematurity complications whose pathogenesis includes the oxidative stress among etiological factors, such as bronchopulmonary dysplasia (BPD), intraventricular hemorrhage (IVH), and retinopathy of prematurity (ROP) [6–8]. Tokuriki et al. showed a correlation between high levels of COHb and moderate-to-severe BPD during the early postnatal period [9]; Bednarczuk et al. found high levels of COHb during the first week of life in preterm infants who develop BPD and IVH [10]; Tagliaferro et al. were not able to demonstrate a correlation between COHb levels and the risk for BPD within the first 2 weeks of life, although COHb levels were higher in infants with BPD [11]; we recently reported that COHb measured in the first week of life can predict the risk for BPD and IVH in very preterm infants [12].

Furthermore, Varal and Dogan demonstrated an increase in COHb levels in preterm infants who develop late-onset sepsis (LOS) and its decrease in association to an effective antibiotic therapy [13]. However, McArdle et al. were not able to find this correlation [14], and, therefore, further studies to confirm or confute the correlation between COHb and LOS in preterm infants could be useful.

Thus, on the basis of previous considerations, we hypothesized that COHb blood levels measured the days before the onset of LOS may be correlated with the risk of its development. To evaluate this hypothesis, we planned the present retrospective study in a cohort of very preterm infants in whom the correlation between COHb levels and the risk of LOS was evaluated.

## Methods

This retrospective observational study ran from November 2017 to October 2021 in the third-level neonatal intensive care unit of the Careggi University Hospital of Florence after approval by the Pediatric Tuscany local ethics committee. Inborn infants with gestational age < 30 weeks who developed LOS were enrolled in the study. Exclusion criteria were proven or suspected hemolytic disorder, major congenital malformations, chromosomal syndromes, inherited metabolic disorders, fetal hydrops, and death within the 36th week of post-menstrual age. A historical control group was constructed by enrolling the infant born after each infant in the study group in a 1:1 ratio using the same inclusion and exclusion criteria except no LOS.

COHb blood levels were measured in blood samples using a blood gas analyzer (ABL800, Radiometer Medical ApS, Brønshøj, Copenhagen, Denmark). COHb levels were expressed as a percentage of the total hemoglobin (Hb).

Arterial or capillary blood gas analyses reported in the electronic records were reviewed and for each infant in the LOS group COHb levels measured on the 1st and 7th day of life, the day of diagnosis of the first episode of LOS, and 3, 2, and 1 days before and 1 and 4 days after the onset of LOS were recorded. Moreover, COHb levels measured on the 1st and 7th day of life and on the same days of life as the infant in the study group born before him when LOS was diagnosed were also recorded for each infant in the control group. When more than one daily blood gas analysis was available, the mean daily COHb level was calculated and recorded.

For each infant we recorded gestational age, birth weight, type of delivery, antenatal steroids, chorioamnionitis, need and duration of oxygen-therapy, noninvasive, and invasive ventilation (mechanical ventilation, MV), age at diagnosis of LOS, occurrence of BPD, sepsis, IVH, ROP, necrotizing enterocolitis (NEC) > 2 grade, death, and duration of hospitalization.

The diagnosis of sepsis was based on clinical and laboratory data (total neutrophil count, C-reactive protein, procalcitonin) confirmed by the presence of at least one positive blood or liquor culture. Antibiotic prophylaxis of early onset sepsis was performed by intravenous administration of penicillin combined with an aminoglycoside discontinued after 48 h of negative blood culture in infants with stable clinical conditions. Antibiotic therapy of LOS was started with intravenous administration of vancomycin combined with an aminoglycoside and eventually adjusted based on the antibiogram. BPD was defined based on the classification of Jobe and Bancalari [15]. The adapted classification of Papile et al. was used to classify the severity of IVH [16]. The diagnosis of NEC was made according to Bell’s criteria [17]. The ROP was evaluated in accordance with the International Classification of ROP [18].

### Primary and secondary endpoints

The primary endpoint of our study was to assess the possible correlation between COHb levels and the risk for LOS development. Secondary endpoint was the comparison of COHb levels in infants who developed Gram-positive sepsis with those in infants who developed Gram-negative sepsis to rule out possible differences.

### Statistical analysis

It has been previously reported that infants with LOS have a COHb level about 30% higher than infants without LOS [13]. Therefore, we calculated that a sample size of 32 infants in each group was needed to detect as statistically significant a difference of 30% of COHb level between the groups, with 80% power at 0.05 level.

Clinical characteristics of infants were reported as mean and standard deviation or rate and percentage. Missing data were replaced with an estimated value based on available information by mean imputation. The Student’s “*t*” test for parametric continuous variables, the two sample Wilcoxon rank-sum test for non-parametric continuous variables, and the *Χ*[2] test for categorical variables were used to compare clinical characteristics of infants in the study and historical group. A *P* < 0.05 was considered statistically significant. Changes of COHb levels before and after LOS were compared by repeated-measures analysis of variance (ANOVA).

We planned to perform logistic regression analyses to assess the possible correlation between the risk of LOS and variables that at univariate analysis were different between the groups (*P* < 0.200) excluding those which were found to be collinear by calculating variance inflation factors (VIF). Therefore, a multivariable logistic regression analysis was performed to evaluate the possible independent effect of sex, need for MV, and COHb level measured 3, 2, and 1 days before the onset of LOS.

To analyze the predictive value of COHb level measured 2 days before the onset of LOS on its occurrence, we used ROC (receiver operating characteristic) curve analysis. The test’s ability to classify patients as those who will develop LOS or not is represented by the area under the ROC curve (AUC). The cut-off point of the ROC curve indicates the COHb level that gives the most true and least false indications of LOS development and, therefore, has the best predictive power.

## Results

We studied 100 preterm infants, 50 included in the LOS and 50 in the no LOS group. The clinical characteristics of the two groups were similar with the exception of occurrence of MV which was more frequent (66 vs. 38%, *P* = 0.005) and longer (17.4 ± 18.9 vs. 8.3 ± 8.3 days, *P* = 0.047) in infants who developed LOS (Table 1). Infants in the LOS group developed it at 16 ± 12 days of life. Twenty-nine (58%) patients had positive blood culture for coagulase-negative staphylococci (CNS), five (10%) for *Staphylococcus aureus*, six (12%) for *Klebsiella* spp., four (8%) for *Escherichia coli*, 3 (6%) for *Enterobacter* spp., and one (2%) each for *Streptococcus gallolyticus* spp*.*, *Serratia* spp., and *Enterococcus* spp. No patient developed meningitis.

**Table 1:** Clinical characteristics and carboxyhemoglobin (COHb) blood levels in infants with or without late-onset sepsis (LOS). Mean (± SD) or rate (%) or median and (range)

	LOS (*n* = 50)	No LOS (*n* = 50)	*P*
Gestational age (wks)	26.6 ± 1.7	26.7 ± 1.6	0.901
Birth weight (g)	885 ± 243	905 ± 219	0.671
Female	22 (44)	30 (60)	0.161
Antenatal steroids	44 (88)	45 (90)	1.000
Cesarean section	29 (58)	29 (58)	1.000
Maternal clinical chorioamnionitis	2 (4)	3 (6)	1.000
Oxygen-therapy Duration (d)	50 (100) 47.2 ± 20.2	50 (100) 47.1 ± 12.9	1.000 0.977
Noninvasive ventilation Duration (d)	50 (100) 28.7 ± 15.2	50 (100) 31.2 ± 19.1	1.000 0.471
Mechanical ventilation Duration (d)	33 (66) 17.4 ± 18.9	19 (38) 8.3 ± 8.3	0.005 0.047
Age at diagnosis of LOS (d)	16 ± 12	NA	NA
Intraventricular hemorrhage	14 (28)	9 (18)	0.342
Bronchopulmonary dysplasia	16 (32)	13 (26)	0.651
Retinopathy of prematurity	13 (26)	9 (18)	0.469
Necrotizing enterocolitis	8 (16)	4 (8)	0.218
Death	2 (4)	0 (0)	0.495
Duration of hospital stay (d)	91.8 ± 37.6	91.9 ± 28.9	0.988

Mean COHb levels measured at the scheduled timepoints were similar between the groups with the exception of COHb levels measured 2 days before the diagnosis of LOS which were higher in the LOS than in the control group (1.61 ± 0.43% vs. 1.46 ± 0.30, *P* = 0.038) (Table 2). Moreover, repeated-measures analysis of variance demonstrated that changes of COHb levels before and after LOS were statistically significant (*P* < 0.001), while we did not find differences in COHb levels in infants with Gram-positive (*n* = 36; 72%) or Gram-negative (*n* = 14; 28%) LOS (Table 3).

**Table 2:** Carboxyhemoglobin (COHb) blood levels (%) in infants with or without late-onset sepsis (LOS). Mean (± SD)

	**LOS** **(*n* = 50)**	**No LOS** **(*n* = 50)**	***P***
**1st day of life**	1.51 ± 0.28	1.47 ± 0.25	0.453
**7th day of life**	1.55 ± 0.39	1.54 ± 0.41	0.901
3 days before LOS*	1.53 ± 0.40	1.43 ± 0.36*	0.173
2 days before LOS*	1.61 ± 0.43	1.46 ± 0.30*	0.038
1 day before LOS*	1.57 ± 0.41	1.44 ± 0.39*	0.122
Day of diagnosis of LOS*	1.57 ± 0.34	1.51 ± 0.35*	0.350
1 day after diagnosis of LOS*	1.50 ± 0.46	1.41 ± 0.33*	0.277
4 days after diagnosis of LOS*	1.52 ± 0.38	1.42 ± 0.41*	0.195

^*^Mean COHb levels measured on the same days of life as corresponding infants in the LOS group

**Table 3:** Carboxyhemoglobin (COHb) blood levels (%) in infants with Gram-positive or Gram-negative late-onset sepsis (LOS). Mean (± SD)

	**Gram-positive LOS** **(*n* = 36)**	**Gram-negative LOS** **(*n* = 14)**	***P***
3 days before LOS	1.58 ± 0.43	1.47 ± 0.53	0.451
2 days before LOS	1.63 ± 0.43	1.54 ± 0.46	0.518
1 day before LOS	1.61 ± 0.43	1.42 ± 0.34	0.145
Day of diagnosis of LOS	1.59 ± 0.35	1.46 ± .033	0.237
1 day after diagnosis of LOS	1.54 ± 0.49	1.37 ± 0.26	0.226
4 days after diagnosis of LOS	1.51 ± 0.34	1.59 ± 0.47	0.507

**Fig. 1:**
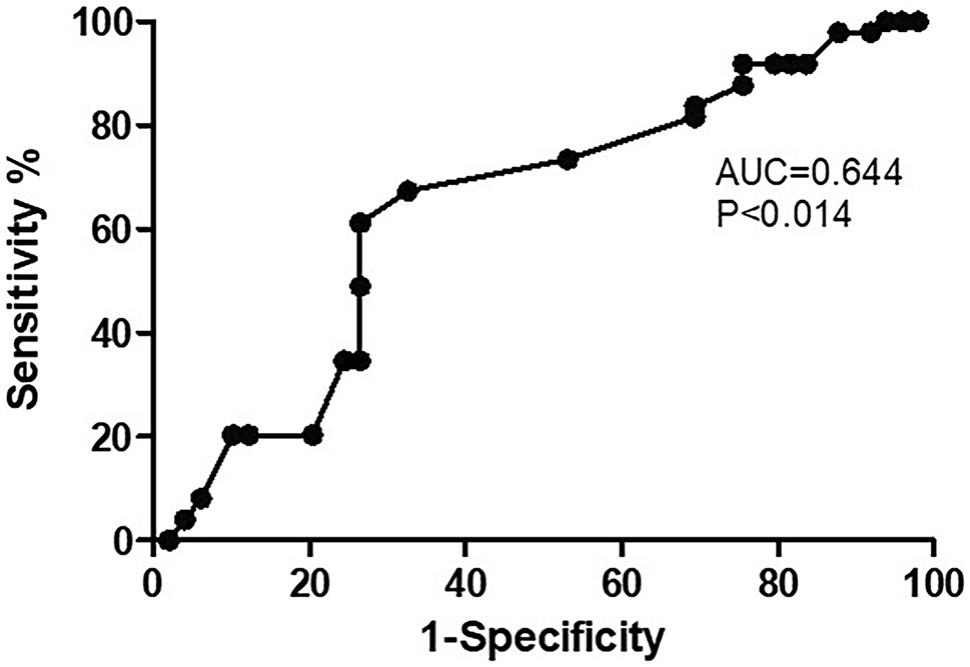
ROC curve analysis for COHb blood levels measured 2 days before the diagnosis of late-onset sepsis (LOS). The area under the curve is 0.644, 95% CI 0.532–0.755. The COHb levels plotted curve indicated 1.55% as the best predictive threshold with a sensitivity of 70% and a specificity of 70%. The ROC curve discriminates preterm infants with LOS from preterm infants without it

Logistic regression analysis showed that a higher level of COHb 2 days before the diagnosis of LOS increases the risk for developing LOS (OR 12.150, 95% Cl 1.311–12.605; *P* = 0.028), as well as the need for MV (OR 3.217, 95% Cl 1.247–8.302; *P* = 0.016). In ROC analysis, COHb measured 2 days before the diagnosis significantly predicts LOS, with an AUC of 0.644 and 95% CI 0.532–0.755 (*P* < 0.014), showing the best prognostic cutoff point at COHb = 1.55%, with a sensitivity of 70% and a specificity of 70% (Fig. 1).

## Discussion

In this study, we assessed the hypothesis that COHb blood levels can predict the development of LOS in very preterm infants when measured in the days immediately before its onset. We found that COHb blood levels significantly increased before the diagnosis of LOS and that COHb level measured 2 days before the diagnosis of LOS was higher in infants in the study than in the control group. Regression analysis confirmed that COHb levels measured 2 days before the diagnosis of LOS were independently correlated with the risk for LOS. Moreover, we found that a COHb level of 1.55% measured 2 days before the diagnosis of LOS is the best predictive threshold for LOS with a sensitivity of 70% and a specificity of 70%. These results are promising because, if confirmed, they suggest for the first time that an easy-to-measure biomarker such as COHb may predict an increased risk of LOS and allow for rigorous monitoring and early diagnosis in high-risk preterm infants.

The correlation between increased level of COHb and the risk of LOS development can be explained by the association between an increased level of COHb and oxidative stress [3–5], since an increase of HO induction and activity (and consequently an increased synthesis of CO) represents a response to oxidative stress [19] which is involved in detrimental pathways activated during neonatal sepsis [20]. In fact, the antioxidant effect of HO can be exerted in vivo by mechanisms other than bilirubin formation, including the multiple ways by which CO modulates inflammatory processes, such as the reduction of neutrophil adhesion and extravasation [21], the reduction of histamine release from mast cells and human basophils [22, 23], inhibition of the expression of proinflammatory cytokines such as tumor necrosis factor-α and IL-1β, and an increase of anti-inflammatory cytokine IL-10 [24].

Previous studies showed an increase of endogenous CO production in adult [25] and pediatric [4] patients with septic shock. Moreover, Varal and Dogan [13] studied a cohort (*n* = 207) of preterm infants with gestational age < 37 weeks and found that COHb level measured at the time of LOS diagnosis was higher than that measured in the first week of life and 1 week after LOS diagnosis. They calculated that at a cut off level of 1.35% COHb had a sensitivity of 56% and a specificity of 90% in confirming LOS [13]. Thus, while we showed that COHb can be predictive of an increased risk of LOS, Varal and Dogan demonstrated an association between increased values of COHb and the diagnosis of LOS, as well as other more accurate biomarkers of sepsis, such as C-reactive protein (CRP) and procalcitonin (PCT). In fact, they did not measure COHb level in the days preceding LOS and did not perform a multivariate data analysis to individuate factors independently associated with LOS in their population [13]. Also, McArdle et al. investigated the possible correlation between COHb level and the development of sepsis in preterm infants, but although they reported that in some patients high levels of COHb did occur during episodes of sepsis, they did not find a consistent temporal pattern between COHb changes and the start of the episode of sepsis [14]. However, it is not easy to interpret the results of this study because they may depend on the small size of population (*n* = 25) and data useful to support the reliability of the results, such as the number and timing of COHb level measurements, were not reported [14].

We compared changes of COHb levels in infants who developed Gram-positive or Gram-negative sepsis, but we did not find differences confirming the results of previous studies [13].

Limitations of our study include its retrospective design but the size of our population was sufficient to have the expected statistical power. Moreover, our data have recently been collected in a homogeneous population over a short period of time; and the statistical analysis was rigorous. Therefore, we are confident that our results are accurate and reliable. We calculated that the best predictive cut off of COHb measured 2 days before LOS has a sensitivity and specificity of 70% which is sub-optimal. However, further larger studies could help improve the accuracy of this biomarker, and, in any case, finding an unexpected increase in COHb should represent, in our opinion, only a warning and an indication for an even more careful clinical evaluation of the patient.

In conclusion, we found that increased levels of COHb measured 2 days before the diagnosis of LOS were independently correlated with an increased risk for LOS. Moreover, we found that a COHb level of 1.55% measured 2 days before the diagnosis of LOS is the best predictive threshold for LOS with a sensitivity of 70% and a specificity of 70%. If further studies confirm our results and possibly improve the accuracy of this easy-to-measure biomarker, neonatologists will have another tool that will aid in the monitoring and early diagnosis of sepsis in high-risk preterm infants.

## Data Availability

Data are available on reasoned request.

## Abbreviations

BPD: Bronchopulmonary dysplasia
COHb: Carboxyhemoglobin
Hb: Total hemoglobin
HO: Heme oxygenase
IVH: Intraventricular hemorrhage
LOS: Late-onset sepsis
NEC: Necrotizing enterocolitis
ROP: Retinopathy of prematurity
